# Supplementary material for: Antigenic Determinant of Helicobacter pylori FlaA for Developing Serological Diagnostic Methods in Children
Source: Pathogens. 2022 Dec 15;11(12):1544. doi: 10.3390/pathogens11121544 (PMC9782684; doi:10.3390/pathogens11121544)
Supplement: Supplementary file 1 [file pathogens-11-01544-s001.zip › Supplementary Data S1.pdf]

1 -ATGGCTTTTCGGGTCAATACAAATATCAATGCGATGAATGCGCATGTGCAATCCGCACTCACTCAAAATGCGCTTAAAACTTCATTGGAGAGATTGAGTTCAGGTTTAAGGATT

M A F R V N T N I N A M N A H V Q S A L T Q N A L K T S L E R L S S G L R I

115 -AATAAAGCGGCTGATGACGCATCAGGTATGACGGTGGCGGATTCTTTGCGTTCACAAGCGAACAGTTTGGGTCAAGCGATTGCCAACACGAATGATGGCATGGGGATTATCCAG

N K A A D D A S G M T V A D S L R S Q A N S L G Q A I A N T N D G M G I I Q

229 -GTTGCGGATAAGGCTATGGATGAGCAGTTGAAAATCTTAGACACCGTTAAGGTTAAAGCGACTCAAGCGGCTCAAGATGGGCAAACTACGGAATCTCGTAAAGCGATTCAATCT

V A D K A M D E Q L K I L D T V K V K A T Q A A Q D G Q T T E S R K A I Q S

343 -GACATCGTTCGTTTGATTCAAGGTTTAGATAATATCGGTAACACGACTACTTATAACGGACAAGCGTTATTGTCTGGTCAATTCACTAACAAAGAATTCCAAGTAGGGGCTTAT

D I V R L I Q G L D N I G N T T T Y N G Q A L L S G Q F T N K E F Q V G A Y

457 -TCTAACCAAAGCATTAAGGCTTCTATCGGCTCTACCACTTCGGATAAAATCGGTCAGGTTCGTATCGCTACAGGTGCGTTAATCACGGCTTCTGGGGATATTAGCTTGACTTTT

S N Q S I K A S I G S T T S D K I G Q V R I A T G A L I T A S G D I S L T F

571 -AAACAAGTGGATGGCGTGAATGATGTAACTTTAGAGAGCGTGAAAGTCTCTAGTTCAGCAGGCACGGGGATTGGTGTGTTAGCAGAAGTGATCAACAAAAACTCTAACCGAACA

K Q V D G V N D V T L E S V K V S S S A G T G I G V L A E V I N K N S N R T

685 -GGCGTTAAGGCTTATGCGAGCGTTATCACCACGAGCGATGTGGCGGTCCAGTCAGGAAGTTTGAGTAATTTAACCTTAAATGGGATCCATTTGGGTAATATCGCAGATATTAAG

G V K A Y A S V I T T S D V A V Q S G S L S N L T L N G I H L G N I A D I K

799 -AAAAACGACTCAGACGGACGATTGGTTGCAGCGATCAATGCGGTTACTTCAGAAACCGGCGTGGAAGCTTATACGGATCAAAAAGGGCGCTTGAATTTGCGCAGTATAGATGGT

K N D S D G R L V A A I N A V T S E T G V E A Y T D Q K G R L N L R S I D G

913 -CGTGGGATTGAAATCAAAACCGATAGTGTCAGTAACGGGCCTAGTGCTTTAACGATGGTTAATGGCGGTCAGGATTTAACAAAAGGCTCTACAAACTACGGAAGGCTTTCTCTC

R G I E I K T D S V S N G P S A L T M V N G G Q D L T K G S T N Y G R L S L

1027-ACACGCTTAGACGCTAAGAGTATCAATGTGGTTTCGGCTTCTGACTCACAGCATTTAGGTTTCACAGCGATTGGTTTTGGGGAATCTCAAGTGGCAGAAACCACGGTGAATTTG

T R L D A K S I N V V S A S D S Q H L G F T A I G F G E S Q V A E T T V N L

1141-CGCGATGTTACCGGGAATTTTAACGCTAATGTCAAATCAGCCAGTGGCGCGAACTATAACGCTGTCATCGCTAGCGGTAATCAAAGCTTGGGAGCTGGGGTTACAACCTTAAGA

R D V T G N F N A N V K S A S G A N Y N A V I A S G N Q S L G A G V T T L R

1255-GGCGCGATGGTGGTGATTGATATTGCCGAGTCTGCGATGAAAATGTTGGATAAAGTCCGCTCTGATTTAGGTTCTGTGCAAAATCAAATGATTAGCACCGTGAATAACATCAGC

G A M V V I D I A E S A M K M L D K V R S D L G S V Q N Q M I S T V N N I S

1369-ATCACTCAAGTGAATGTTAAAGCGGCTGAATCTCAAATTAGGGATGTGGATTTTGCTGAAGAGAGTGCGAATTTCAATAAAAACAACATTTTGGCACAATCAGGTAGCTATGCG

I T Q V N V K A A E S Q I R D V D F A E E S A N F N K N N I L A Q S G S Y A

1483-ATGAGTCAAGCCAATACCGTCCAACAAAATATCTTAAGGCTTTTAACTTAG

M S Q A N T V Q Q N I L R L L T *

Supplementary Data S1. FlaA full sequence.
