# Supplementary material for: Antigenic Determinant of Helicobacter pylori FlaA for Developing Serological Diagnostic Methods in Children
Source: Pathogens. 2022 Dec 15;11(12):1544. doi: 10.3390/pathogens11121544 (PMC9782684; doi:10.3390/pathogens11121544)
Supplement: Supplementary file 1 [file pathogens-11-01544-s001.zip › Supplementary Data S2.pdf]

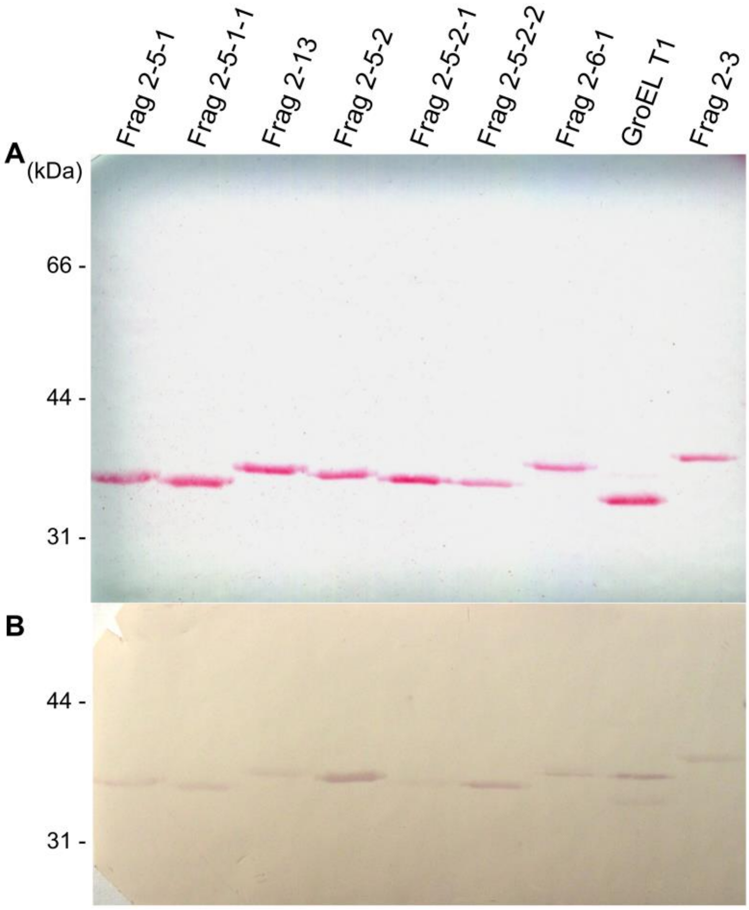

Supplementary Data S2. Immunoblot analysis of the recombinant subfragment proteins of FlaA. (A) Ponceau S staining and (B) Western blot analysis using *H. pylori*-positive pooled sera. Frag 2–13 is a subfragment recombinant protein of FlaA 1237–1347 bp. Because Frag 2–13 was divided into Frag 2–5-1–1 and Frag 2–5-2–1 to analyze the antigen reactivity in the present study, Frag 2–13 has not been explained.
